# Modern Physical Education and Its Influence on Students’ Entrepreneurial Psychology in Sports Universities

**DOI:** 10.3389/fpsyg.2021.751176

**Published:** 2021-12-24

**Authors:** Daliang Zhou, Delong Zhu, Fengrui Zhang, Guangxue Li, Ke Zong

**Affiliations:** ^1^School of PE, Nanjing Xiaozhuang University, Nanjing, China; ^2^School of Management Engineering, Anhui Institute of Information Technology, Wuhu, China; ^3^Trinity College, University of Cambridge, Cambridge, United Kingdom; ^4^College of Physical Education, China West Normal University, Nanchong, China; ^5^College of Physical Education, Zhengzhou University, Zhengzhou, China

**Keywords:** entrepreneurial psychology, physical education, human-computer interaction, Internet+, entrepreneurial motivation

## Abstract

This study aims to explore the entrepreneurial psychology of physical Education (PE) students under the “Internet+”environment, to cultivate and improve the entrepreneurial consciousness of PE students, taking the realization of students’ sense of self-efficacy as an intermediary factor. The new educational technology in modern PE is analyzed first. Specifically, the motion sensing technology based on human-computer natural interaction can be used for training, so that learners can effectively improve their physical skills. Subsequently, the current entrepreneurial situation of PE majors is discussed, with 188 students from Tianjin University of Sport and Guangzhou Sport University selected as research subjects. It is found that 62.2% of students have never been exposed to online entrepreneurship, and they are more afraid of entrepreneurial risks. In terms of entrepreneurial motivation, most students choose to start a business because of “personal ideals,” and only 40 people choose to start a business because of economic factors. There is a significant positive correlation between entrepreneurial self-efficacy and entrepreneurial intention of college students majoring in PE, and the correlation coefficient is 0.488. At present, the teaching mode of sports universities focuses on the teaching of professional courses. However, students generally believe that the professional knowledge learned is not useful for future entrepreneurship. The entrepreneurial self-efficacy of college students tends to be positive, and there are notable differences in the entrepreneurial self-efficacy between boys and girls. The regression analysis of entrepreneurial self-efficacy and entrepreneurial intention of college students shows that entrepreneurial self-efficacy can effectively predict entrepreneurial intention. This research promotes the innovation and development of the sports industry under the background of “Internet+”.

## Introduction

Traditional Physical Education (PE) training is usually boring, making teachers and students suffer burnout. Further, it fails to make individualized training plan (Rapp, 2020). With the emergence of the new education model of “big data+artificial intelligence+education,” the school began to harness the sports information service platform for data collection and accumulation, providing decision-making guidance for education management (Urquiza-Fuentes and Paredes-Velasco, 2017; Kuo et al., 2019). “Internet+sports” refers to the new form and new normal under innovation 2.0. It applies the Internet innovation achievements in sports industry to promote the reform in traditional sports industry chain, market forms and business model, reform the sports industry evolution model and business model, and enhance the innovation, productivity, and core competitiveness of the sports industry, to form a new form of sports industry with Internet+as infrastructure and implementation tools. As information technology progresses rapidly, human-computer interaction has been realized and applied to industry, medicine, smart city construction, physical exercise (Kim et al., 2020). During physical exercise, human-computer intelligent interaction is realized through the detection of sports targets using intelligent devices (Hou et al., 2019). The final data of personal training will also be fully recorded by the system, and more scientific exercise suggestions will be given through big data analysis. The PE activities based on somatosensory technology enhance students’ sports experience. Additionally, this technology supports multiple people learning together and enables online and offline interactions, increasing the entertainment in PE.

With the support of human-computer interaction technology, sports majors can better improve their training quality and professional skills. For students in school, the solid professional skills can not only lay the foundation for them to engage in related work after entering the society, but also provide necessary support for students with entrepreneurial ideas in sports-related fields. For example, to establish sports service platform and sports information public account, it is necessary to accumulate professional skills in school. In 2019, the number of university graduates in China exceeded 8.3 million, bringing about a serious employment problem. The Ministry of Education has instructed that the employment and entrepreneurship of graduates is an important aspect of livelihood, as well as a current national plan. The current employment situation for PE majors is not optimistic, because there are few jobs open to them (Kirby et al., 2018).

In the modern education context where various intelligent teaching methods are emerging in an endless stream, the proposal and practice of the “Internet+” plan provides new ideas for college students’ online entrepreneurship. In the study, the intelligent modern PE teaching method based on human-computer interaction is first expounded, especially the motion sensing technology to enhance the interest of PE majors in learning and training. The innovation of this research lies in exploring the application of human-machine interaction technology in PE, which can help students correct their movements. Subsequently, the current entrepreneurship situation of PE majors is investigated, with a new type of online entrepreneurship model proposed to enhance students’ online entrepreneurship willingness under the background of “Internet+.” The study, on the one hand, enhances the ability of sports professional training; on the other hand, it has a certain value to enhance students’ entrepreneurial awareness and expand their horizons.

### Recent Related Works

Even though to be PE teachers in primary and middle schools is a good choice for them, there are not enough jobs for all graduates, leading to fierce competition. Therefore, to help PE majors to start a business is a new hot spot to solve the employment problem (Millar and Doherty, 2018). Hu and Ye (2017) conducted a survey on the entrepreneurial intention of PE majors, and concluded that entrepreneurial self-efficacy is a key cognitive index affecting their entrepreneurial intentions. Hence, educational institutions should encourage graduates to start their own businesses to respond to the increasingly fierce employment competition. Holienka et al. (2018) discussed the personality traits, enterprise spirit, and entrepreneurial tendency of PE majors. It was found that they exhibited higher entrepreneurial tendency and enterprise spirit. Consequently, effective measures can be taken to strengthen students’ entrepreneurial intentions.

It is difficult for inexperienced graduates to start a business. They are under great psychological pressure. Therefore, extensive research on college students’ entrepreneurial psychology will help them to carry out entrepreneurial activities (Yueh et al., 2020). Based on the market distribution of college students’ entrepreneurial assets, Kong and Zhao (2020) applied prospect theory to describe the psychological characteristics of college students’ entrepreneurial assets loss. Then, according to the adjustment cost of the investment portfolio, they constructed the college students’ investment portfolio model under entrepreneurship dependence and loss psychology, and evaluated the asset return characteristics and effectiveness of the model (Kong and Zhao, 2020). Zhao et al. (2020) discussed the influence of psychological capital on entrepreneurial intention of Chinese college students. Entrepreneurship must be analyzed from the perspective of personal characteristics, especially considering the differences between personal characteristics and entrepreneurial capital.

In this study, PE students are selected as the research subjects. From the perspective of entrepreneurship psychology, a new model of network entrepreneurship is proposed to improve the willingness of college students to network entrepreneurship under the background of “Internet+.”

## Materials and Methods

### Characteristics and Functions of Human-Computer Interaction Technology in Physical Education

In PE and training, on the one hand, the teachers observe students through the eyes, ears, and other sense organs; on the other hand, they process the information received to make decisions to make dynamic adjustments to the training content. Nowadays, more and more new educational methods are emerging resulting from the development and popularization of electronic technology (Chamberlain et al., 2017). While changing the teaching form, it has also brought a change in thinking mode. With the digital automatic training system taken as an example, the student’s training process is recorded digitally through motion capture technology for accurate modeling and calculation, followed by training effects analysis and targeted training guidance (De Marsico and Fogli, 2017). Moreover, automatic evaluation and training based on data information can reduce the dependence on teachers.

In PE, motion sensing control technology can be applied to the actual training of students to realize real-time recognition of students’ sense organs and movements using electronic equipment. During the process, the user’s movements are fed back based on the predetermined motion sensing mode, so that students experience the training more intuitively (Bolgova et al., 2020).

**FIGURE 1:**
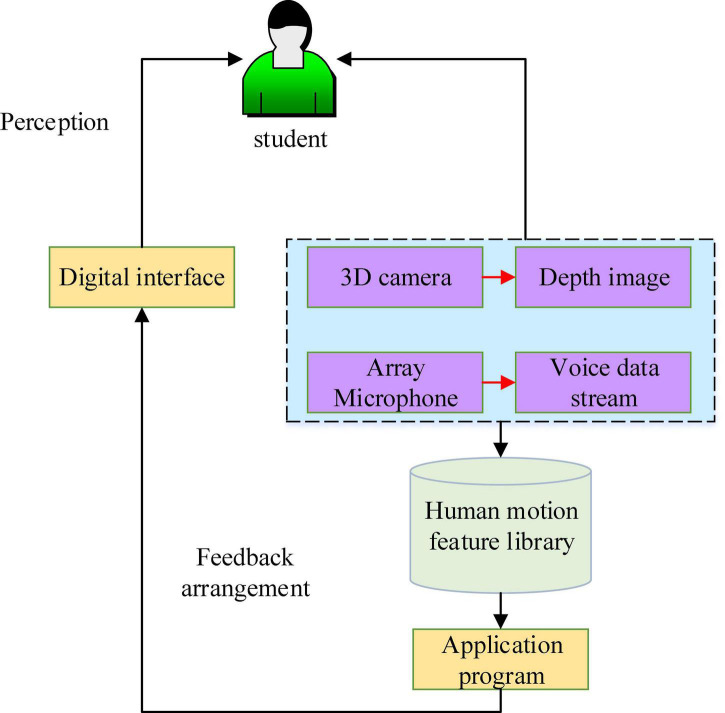
Motion sensing technology based on human-computer interaction in Physical Education (PE).

Kinect technology, which uses optical technology to recognize human joint points, identifies the sensory movements of students through corresponding motion sensing equipment to obtain the data stream of color, depth, and sound (Chang et al., 2020). Through data analysis and comparison with preset human movements, problems existing in training are put forward, which are finally fed back to students in a visual or auditory form on the interface of the system. The application of motion sensing technology based on human-computer interaction in PE is shown in Figure 1. Kinect detects learners’ sensory movements using corresponding somatosensory peripherals, 3D cameras, array microphones, and other equipment, and obtains the learner’s data stream. Then, the data is compared with built-in human movements to judge whether the learner’s movements are standard. Next, the results are fed back to the learner on the system interface, either visually or acoustically, to improve his/her learning skills.

Kinect uses a natural human-computer interaction interface, so that human interacts with the computer through the preset movements. In addition to regular body movements, the Kinect somatosensory system can also capture the user’s emotions through subtle changes in facial expressions. The improved accuracy of the depth sensor leads to more detailed small movements capture, even the movements of each finger. The bone tracking system can not only track joints, but also detect the strength of the movement (Palacios-Navarro et al., 2015; Yao et al., 2015; Cao et al., 2019). The sound sensor can more accurately and quickly recognize the content and direction of the voice. This kind of human-computer interaction based on motion sensing technology is very attractive to students. They can talk to the computer in front of the camera during physical exercise to coordinate the body.

**FIGURE 2:**
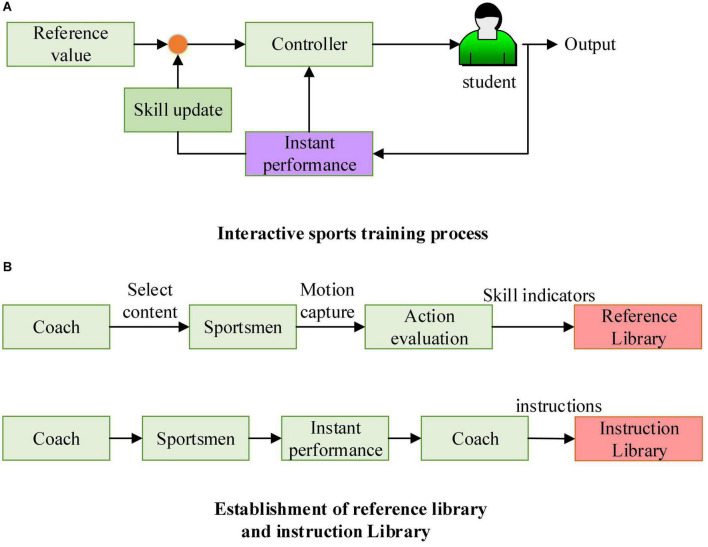
Interactive sports training process and the establishment of the corresponding reference library and instruction library. **(A)** Interactive sports training process. **(B)** Establishment of reference library and instruction library.

In the initial stage of interactive training, students set a goal based on the reference library. Subsequently, students choose a standard test of a certain difficulty level according to their own situation, which is to understand their skill level (Berns et al., 2016; Castaneda and Cho, 2016). The system can calculate suitable training difficulty based on the student’s skill level, the expected level, and other information. If the student reaches the standard, the training difficulty and progress will be updated; if the student fails to reach the standard, the system will select the instruction with the highest similarity to the current training difficulty to update the training content. During the entire physical training process, if students feel that there is deviation from their skill level, they can take the standard test again to correct each skill index. The complete interactive sports training process and the establishment of the corresponding reference library and instruction library are shown in Figure 2.

### Entrepreneurship Situation Under the Background of “Internet+”

From the content of the previous section, it is noted that the somatosensory technology based on human-computer interaction has shown strong vitality and diversified development trends in PE. It is suitable to use somatosensory technology for PE in ordinary classrooms, rooms, playgrounds, and open spaces. Through continuous updating of learning content, it increases the interest of learners. At the same time, learners can choose suitable space and time for PE according to their own situation. Under a more advanced teaching mode, the learners in PE colleges can have a better understanding of their own majors and better cognition of their own skills. With the advent of the “Internet+” era, students can be more exposed to the diversified state of employment in the social environment. On the basis of the comprehensive development of their professional-level skills, they can broaden their entrepreneurial ideas and give full play to their personal value.

“Internet+” is a new form of the integrated development of the Internet and traditional industries. It upgrades traditional industrial structure, production methods, and operating modes, thereby creating new business value (Yan et al., 2018). The era of “Internet+” eliminates the original information asymmetry pattern, enabling resources to be utilized and shared to the maximum (He and Ma, 2020; Cooke and Xiao, 2021). Also, the “Internet+” creates new jobs for college students. However, the new business model also puts forward higher requirements on the comprehensive quality of practitioners, and information technology talents are popular among various enterprises.

**FIGURE 3:**
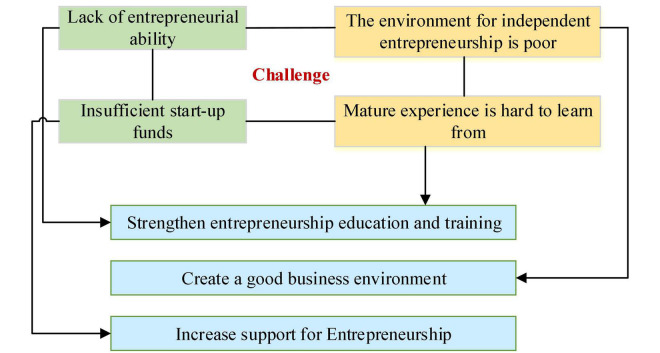
The challenges faced by college students during entrepreneurship and the corresponding solutions under “Internet+” context.

Accompanied by changes in thinking mode, production, and consumption, Internet technology is constantly expanding the entrepreneurial space of college students. Under “mass entrepreneurship and innovation” atmosphere (Moreno, 2016; Cheng et al., 2018; Xiao et al., 2020). colleges and universities can cultivate students’ entrepreneurial awareness by organizing entrepreneurial competitions, which will help students better adapt to the ever-changing job market in the future. For students who decide to start a business, the government has formulated corresponding preferential policies, and the school can also provide venues or financial support. In the “Internet+” era, college students can use Internet finance to achieve rapid entrepreneurship, such as opening a store online, or selling products through APP or WeChat official account, or designing a website for product marketing (Liao, 2020). However, college students face many new challenges in the process of innovation and entrepreneurship, as shown in Figure 3. First, college students have just entered the society. Although they have strong professional ability, they have insufficient social resources and entrepreneurial ability. Students in the early stage of entrepreneurship cannot effectively master the complex and changeable market environment due to lack of rational analysis, which directly affects their investment decisions. Second, although the government and schools have set up entrepreneurial funds for college students, there are relatively high thresholds and complicated procedures. Third, although the government formulates relevant policies to provide a favorable external environment for college students’ self-employment, the implementation of the policy is still in the preliminary exploration stage. There is a lack of a complete set of policies and regulations to support college students’ entrepreneurship, which can provide assistance to college students in in finance, intellectual property protection, and other aspects. Finally, “Internet+” is to provide users with new experiences and new services. Under such new business models, there is a lack of mature experience and operational management models college students can refer to.

### Requirements on the Psychological Quality of Starting a Business for Physical Education Majors

To start a business is a very complex systematic project, and the success of entrepreneurship is the result of multiple factors. Psychological quality plays an important role in the comprehensive quality of human beings (Wu and Wu, 2017). Good psychological quality is the foundation to achieve the ultimate success. Therefore, there are higher requirements put forward on college students’ psychological quality (Apriana et al., 2019; Vodã and Florea, 2019). In the face of complex changes and fierce competition, entrepreneurs should flexibly adjust themselves and adapt to changes. In the tide of the market economy, opportunities and risks coexist (Wu et al., 2020). A greater scale of business brings greater risks and psychological burden. Entrepreneurs should be highly adaptable, be good at summarizing experience, and be able to make appropriate adjustments in a timely manner.

In the study, the basic psychological qualities necessary for PE majors to start a business are summarized as follows. First, students should have motivations for entrepreneurship. From the perspective of psychology, a stronger motivation contributes to stronger desire, conducive to improvement of the personal comprehensive ability and thus increasing the success rate. Second, students should have high spirits for entrepreneurship. Faced with obstacles during the entrepreneurship, students act with great determination to overcome difficulties. Third, students should be confident in themselves. The rapidly changing market environment prompts entrepreneurs to continuously adjust their development strategies. In this process, they may make a wrong decision. At this time, they should judge themselves objectively, and face the next challenges more actively. Fourth, students should show great perseverance during the process. One’s self-ability is embodied by hos willpower to a certain extent. Driven by willpower, people can effectively manage thoughts and emotions, and will restrain temporary desires for long-term benefits.

### Research Design and Research Subjects

Based on entrepreneurial psychology, the entrepreneurship willingness and influencing factors of PE majors are investigated. The questionnaire is initially designed, and then modified by experts. The reliability of the questionnaire is tested using Cronbach’s coefficient test, and α = 0.83 > 0.8 indicates that the questionnaire is relatively stable. The KMO of the questionnaire validity test is 0.85, indicating that this questionnaire is suitable for factor analysis. The validity of the questionnaire is tested by experts, who generally believe that the content of the questionnaire is highly effective.

The final designed questionnaire consists of four parts. The first part is the basic information of the survey object, including age, gender, entrepreneurial education experience, and other information. The second part is a survey on college students’ entrepreneurial motivation and entrepreneurial attitude. The third part is a survey on entrepreneurial intentions using a 5-item scale compiled by Chen et al. (1998). Likert’s 5-point method is adopted, and a score of 1 to 5 means from “totally disagree” to “totally agree.” The fourth part is the entrepreneurial self-efficacy survey using the entrepreneurial self-efficacy scale developed by Jill and Robert. The scale includes four parts: opportunity recognition efficacy, relationship efficacy, management efficacy, and risk tolerance efficacy. A higher value indicates better entrepreneurial self-efficacy (Jill and Robert, 2005).

The research subjects are college students from Tianjin University of Sport and Guangzhou Sport University, with 100 questionnaires distributed. The questionnaires are distributed on the spot after telephone contact. In Tianjin University of Sport, there are 95 valid questionnaires. In Guangzhou Sport University, there are 93 valid questionnaires. The SPSS22.0 software is used to conduct basic analysis and statistics on the survey results of the questionnaire, with the relationship between entrepreneurial self-efficacy and entrepreneurial intention analyzed.

## Results and Discussion

### Basic Information of Research Subjects

The basic information of 188 PE majors is statistically analyzed, and the results are shown in Table 1. Among them, there are slightly more boys than girls. They are mainly from 18 to 21 years old, with 70 students in the third year. The third year is an important turning point in college, and senior students basically has formulated their own career or study plans. Only 35.6% of students often participate in entrepreneurship training, which shows that sports universities and universities have organized entrepreneurship training for students, which should be further promoted. Among the 188 subjects, 62.2% of students have never been exposed to online entrepreneurship. On the one hand, their own abilities are limited. On the other hand, due to insufficient support from schools, government, and society, students with Internet entrepreneurship ideas are also afraid of failure.

**TABLE 1:** Basic information of research subjects.

Information	Options	Number of people	Percentage (%)
Gender	Male	98	52.1
	Female	90	47.9
Age	18∼21 years old	158	84.0
	22∼25 years old	25	13.3
	>25 years old	5	2.7
Grade	Freshman	29	15.4
	Sophomore	44	23.4
	Junior	70	37.2
	Senior	45	23.9
Entrepreneurship education or training	Never exposed to it	33	17.6
	Have had a few experiences	88	46.8
	Often participate in it	67	35.6
Network entrepreneurship experience	Never tried	117	62.2
	Have relevant experience	25	13.3
	Have other forms of entrepreneurial experience	36	19.2
	Online entrepreneurship in progress	10	5.3

### Survey on the Entrepreneurship Motivation and Attitude

**FIGURE 4:**
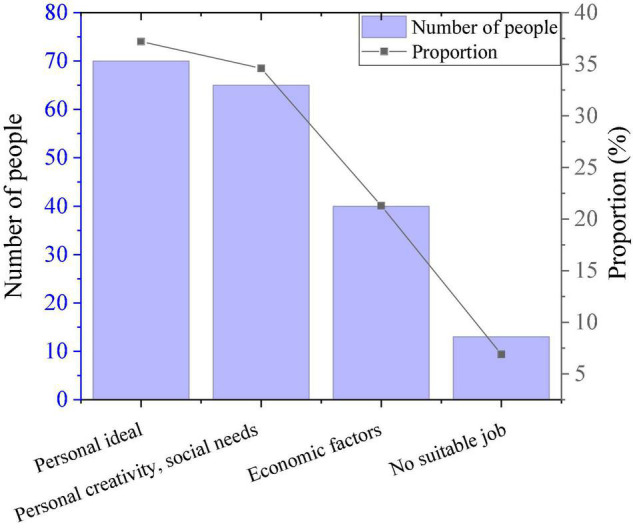
Entrepreneurship motivation survey results.

As shown in Figure 4, 70 subjects choose to start a business because of “personal ideals,” 65 people choose to start a business because “personal creativity meets social needs,” and only 13 people choose to start a business because they don’t have a suitable job. It is not economic factors that rank first among college students’ motivations for online entrepreneurship, but rather to challenge themselves and realize their value in life. Among the 188 subjects, only about 1/3 of the students are willing to spend a lot of time learning about Internet entrepreneurship, while 127 students are unwilling. Obviously, the PE majors don’t hold a positive attitude toward network entrepreneurship.

**FIGURE 5:**
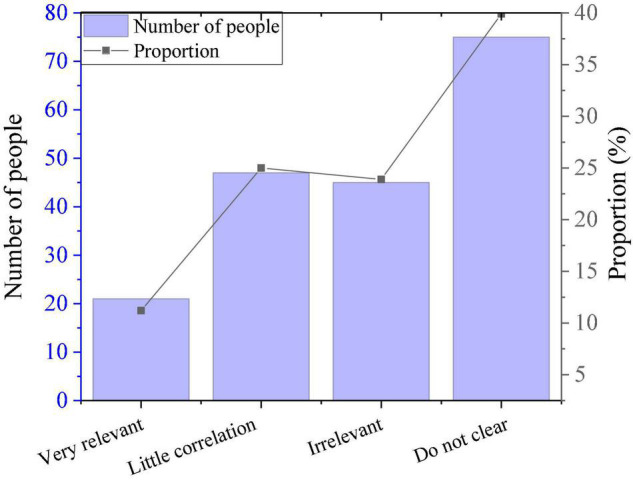
PE majors’ cognition on the relationship between their major and entrepreneurship.

In the study, the subjects are surveyed for their cognition on correlation between their major and entrepreneurship, and the results are shown in Figure 5. Among them, only 21 believe that there is a great correlation between their major and entrepreneurship, 45 think there is no correlation between the two, and 75 are unclear about this. The current teaching model of sports universities is mainly focused on the teaching of professional courses. However, students generally believe that the professional knowledge learned is not useful for future entrepreneurship. The reason may be that there are limitations of the current education model. Therefore, to change this *status quo*, colleges and universities should adjust their educational thinking and change students’ attitudes toward network entrepreneurship.

### A Survey of College Students’ Entrepreneurial Self-Efficacy

**FIGURE 6:**
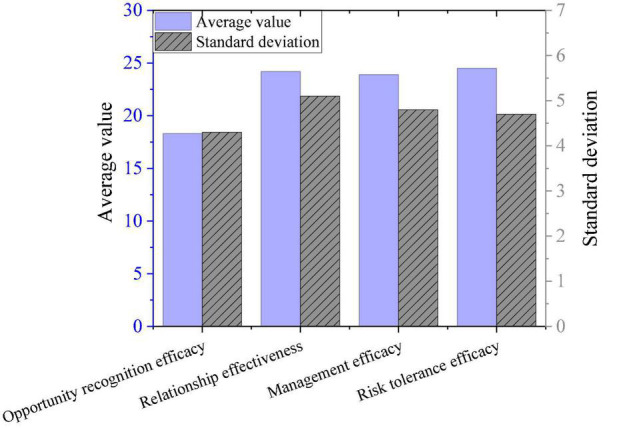
The self-efficacy survey results.

As shown in Figure 6, the entrepreneurial self-efficacy of college students is positive on the whole. The total scale and various scores reveal that the average value of the entrepreneurial self-efficacy exceeds the corresponding median value, indicating that the entrepreneurial self-efficacy of college students tends to be positive. Among them, the risk tolerance efficacy score is the highest, and the opportunity recognition efficacy score is the lowest.

**FIGURE 7:**
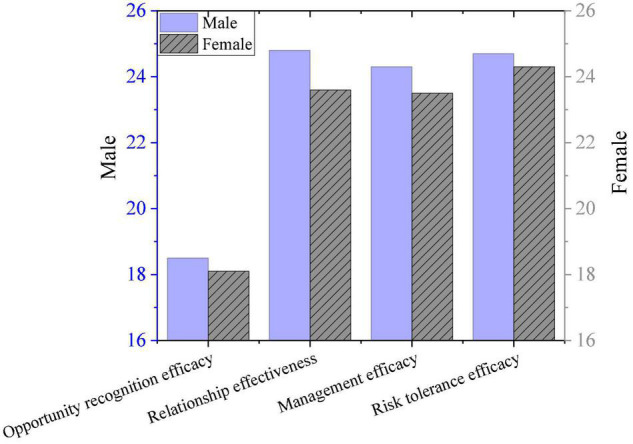
The self-efficacy comparison results.

Figure 7 shows the comparison results of entrepreneurial self-efficacy scores. In terms of homogeneity of variance, there are notable differences in entrepreneurial self-efficacy between boys and girls (*P* < 0.01). The scores of boys on the four efficacy scales are notably higher than those of girls (*P* < 0.001). There are gender differences in the average scores of entrepreneurial environment cognition, the social environment cognition, school entrepreneurial environment cognition, and entrepreneurial policy environment cognition. Since the average of girls is greater than that of boys, it shows that girls are more satisfied with the college environment for entrepreneurship than boys. In terms of entrepreneurial self-efficacy, girls’ average entrepreneurial self-efficacy is higher than that of boys.

### Correlation Between Entrepreneurship Self-Efficacy and Entrepreneurship Intention

Obviously, there is a positive correlation between entrepreneurial self-efficacy and entrepreneurial intention of PE majors, with a correlation coefficient of 0.488. In other words, higher entrepreneurial self-efficacy indicates stronger entrepreneurial intentions. Table 2 is the regression analysis result of entrepreneurial self-efficacy and entrepreneurial intention. The determination coefficient of the regression equation is 0.216, which means that the predictor variable can explain 21.6% of the variance of the dependent variable, and the equation is significant as a whole (*P* = 0.00). It shows that entrepreneurial self-efficacy can effectively predict entrepreneurial intentions. Therefore, colleges and universities should stress the cultivation of students’ entrepreneurial self-efficacy through a series of lectures and seminars on entrepreneurial knowledge, to improve students’ entrepreneurial consciousness, enhance their self-confidence, cultivate their risk awareness, and improve their entrepreneurial willingness. To open psychological lectures and arrange psychological teachers to teach knowledge on entrepreneurial psychology can help would-be entrepreneurs to adjust their physical and mental state, enhance their self-confidence, and pay attention to the development of psychological health.

**TABLE 2:** Regression analysis results of entrepreneurial self-efficacy and entrepreneurial intention.

	Non-standardization factor		Standardization factor	*t*	Significance
	B	Standard error	Beta		
Constant	8.141	0.709	–	11.375	0.000
Entrepreneurial self-efficacy	0.418	0.032	0.468	12.573	0.000

## Conclusion

At present, Internet has found broad applications in various fields. Entrepreneurship in the “Internet+” era means continuous innovation. In the study, the new forms of PE are explored, catalyzed by information technology. Among them, the motion sensing technology with human-computer interaction as the core allows users to interact with the computer, so that they can obtain feedback on the training effects. It is very useful in PE. To understand the entrepreneurial psychology of PE majors under the rapid development of information technology, the entrepreneurial situation of students in two key sports universities is investigated. The “Internet+” provides new ideas of online entrepreneurship. Students in sports universities generally believe that the professional knowledge they have learned is not very useful for future entrepreneurship. The entrepreneurial self-efficacy of college students is positive on the whole. Among them, the risk tolerance efficacy score is the highest, and the opportunity recognition efficacy score is the lowest. The results show that college students’ entrepreneurial self-efficacy tends to be positive. Among them, the risk tolerance efficiency score is the highest, and the opportunity identification efficiency score is the lowest. There is a significant positive correlation between entrepreneurial self-efficacy and entrepreneurial intention of college students majoring in PE, and the correlation coefficient is 0.488.

In response to the current entrepreneurial situation, government departments should improve the legal system for innovation and entrepreneurship under “Internet+,” and heed college students’ entrepreneurial psychological needs, so that relevant policies are adjusted timely to support college students’ entrepreneurship. The colleges and universities need incorporate innovation and entrepreneurship education into the talent training program. At the same time, they should focus on cultivating college students’ Internet thinking and encourage students to start a business through simulation practice. This research provides a theoretical basis for strengthening PE majors’ entrepreneurship intentions under “Internet+.” In the following research, the feasibility of Internet entrepreneurship can be discussed in depth. Due to the uncontrollable factors in the questionnaire distribution in this study, it is inevitable that some people are careless when filling in the data. However, positive measures have been taken to make the results reliable as much as possible. Additionally, this study is still not very proficient in data processing, and training in this area should be strengthened in the future.

## Data Availability Statement

The raw data supporting the conclusions of this article will be made available by the authors, without undue reservation.

## Ethics Statement

The studies involving human participants were reviewed and approved by Gachon University Ethics Committee. The patients/participants provided their written informed consent to participate in this study. Written informed consent was obtained from the individual(s) for the publication of any potentially identifiable images or data included in this article.

## Author Contributions

All authors listed have made a substantial, direct, and intellectual contribution to the work, and approved it for publication.

## Conflict of Interest

The authors declare that the research was conducted in the absence of any commercial or financial relationships that could be construed as a potential conflict of interest.

## Publisher’s Note

All claims expressed in this article are solely those of the authors and do not necessarily represent those of their affiliated organizations, or those of the publisher, the editors and the reviewers. Any product that may be evaluated in this article, or claim that may be made by its manufacturer, is not guaranteed or endorsed by the publisher.
